# Positive correlation between blood glucose and radiotherapy doses to the central gustatory system in Glioblastoma Multiforme patients

**DOI:** 10.1186/s13014-019-1311-3

**Published:** 2019-06-07

**Authors:** Marciana N. Duma, Nadja I. Oszfolk, Tobias Boeckh-Behrens, Markus Oechsner, Claus Zimmer, Bernhard Meyer, Paul T. Pfluger, Stephanie E. Combs

**Affiliations:** 1 Department of Radiation Oncology, Klinikum rechts der Isar, Technical University, Munich, Germany; 2 Faculty of Medicine, Technical University, Munich, Germany; 3 Department of Neuroradiology, Klinikum rechts der Isar, Technical University, Munich, Germany; 4 Department of Radiation Sciences (DRS), Institute of Innovative Radiotherapy (iRT), Helmholtz Zentrum München (HMGU), Oberschleißheim, Germany; 5 Deutsches Konsortium für Translationale Krebsforschung (DKTK), Partner Site Munich, Munich, Germany; 6 Department of Neurosurgery, Klinikum rechts der Isar, Technical University, Munich, Germany; 7 Research Unit NeuroBiology of Diabetes, Helmholtz Diabetes Center, Helmholtz Zentrum München (HMGU), Oberschleißheim, Germany; 8 Department of Radiotherapy and Radiation Oncology, University Hospital Jena, Bachstr. 18, 07743 Jena, Germany

**Keywords:** Glioblastoma, Gustatory system, Radiotherapy, Blood glucose

## Abstract

**Background:**

The aim of this study was to assess the correlations between the levels of blood glucose (BG) and the dose of radiation therapy (RT) to the central gustatory system (GS) in glioblastoma multiforme (GBM) patients.

**Methods:**

Thirty-seven GBM patients with regular blood glucose measurements were investigated retrospectively. 59.5% were female and 40.5% male with a median age of 64.3 years (range 27.4–85.6). Diabetes mellitus type 2 (DM2) history, BG levels and dexamethasone (DEXA) medication were assessed. The analyzed central gustatory structures were: solitary tract and nucleus, ventral posteromedial nucleus of the thalamus, sensory tongue area of the postcentral gyrus, anterior part of the insula, frontal operculum, amygdala, hypothalamus. These structures were delineated on magnetic resonance tomographies (MRIs) registered to planning-CTs. All GS doses were transformed in equivalent doses in 2 Gy fraction (EQD2).

**Results:**

Twenty one patients (56.8%) had at least one BG values over 200 mg/dl during RT. There was a difference between average BG in DM2: 192.8 mg/dl (±24.4) and non-DM2 patients: 145.7 mg/dl (±39.5; *p* = 0.01) but no significant difference in daily DEXA medication – DM2 patients: 7.9 mg/d (±1.9) vs. non-DM2: 9.3 mg/dl (±5.7; *p* = 0.29). The EQD2 Dmean to the total GS was 36.0Gy (±8.6 Gy). There was a tendency for a higher increase in maximum BG values with more radiation dose to the total GS (b = 1.9, R^2^ = 0.103, *p* = 0.06).

**Conclusion:**

BG levels in GBM patients are in direct correlation to the dose of RT applied to the central GS. GBM patients that undergo RT should thus be closely monitored for changes in BG levels during and after the radiation.

## Background

Glioblastoma multiforme (GBM) represents the most common primary malignant tumor of the central nervous system in adults with a low overall survival [1, 2]. Three approaches are used in GBM treatment: neurosurgical resection, radiotherapy (RT) and chemotherapy. The standard of care is surgery followed by RT plus temozolomide. Chemotherapy is given during radiation and six cycles after radiation [3, 4]. For elderly patients hypofractionated RT can be used with or without chemotherapy [5]. The side effects of RT of the brain include hair loss, erythema, fatigue, nausea and vomiting and intracranial oedema [6, 7]. In order to reduce neurological symptoms due to brain oedema, dexamethasone (DEXA) is often used during RT [8, 9]. Notably, it has been reported that patients with brain tumors undergo changes in their blood glucose (BG) during therapy and high BG levels contribute to poor prognosis [10, 11]. Hyperglycaemia in brain tumor patients can result from steroid medication [12], from stress-induced hyperglycaemia caused by acute medical or surgical illness [13] or from damage to the glucose-sensing neurons [14] by tumor or RT. Nonetheless, there is no recommendation yet of regular testing of glucose levels during RT in all patients.

In this study, we aimed to interrogate the impact of RT on BG levels in GBM patients. We specifically aimed to assess whether RT doses to the gustatory system (GS), a major brain network that orchestrates our sensation of sensing, recognizing and interpreting tastes, can have a clinical impact on BG levels during RT. The neurons belonging to the central gustatory system are also known to modulate blood glucose levels. Only tasting food in the oral cavity leads to an increase in circulating insulin, before there is any increase in post absorptive circulating glucose. This is called the cephalic phase of insulin secretion [15, 16]. The GS receives input from sensory taste bud cells on the tongue via afferent fibers from the facial, glossopharyngeal and vagal nerve that build the tractus solitarius and end in the nucleus of tractus solitarius in the brainstem. From here, thalamic fibers project to the ipsi- and contralateral ventral posteromedial nucleus of the thalamus where they switch to neurons projecting to the tongue area of the postcentral gyrus, the frontal operculum and the anterior insula for taste perception. Extrathalamic fibers are connected to the dorsal nucleus of the vagus, hypothalamus and amygdala for visceral and behavioral reflexes and creating emotions [17].

To date, it remains elusive whether RT doses to the central GS have an impact on BG. Since high blood glucose levels are known to be a limitation factor to survival of glioblastoma multiforme patients [10, 11], all causes of blood glucose fluctuation should be understood. Accordingly, in a retrospective study we assessed BG levels in 37 GBM patients with or without diabetes that underwent defined RT doses to central gustatory structures.

## Methods

**Fig. 1:**
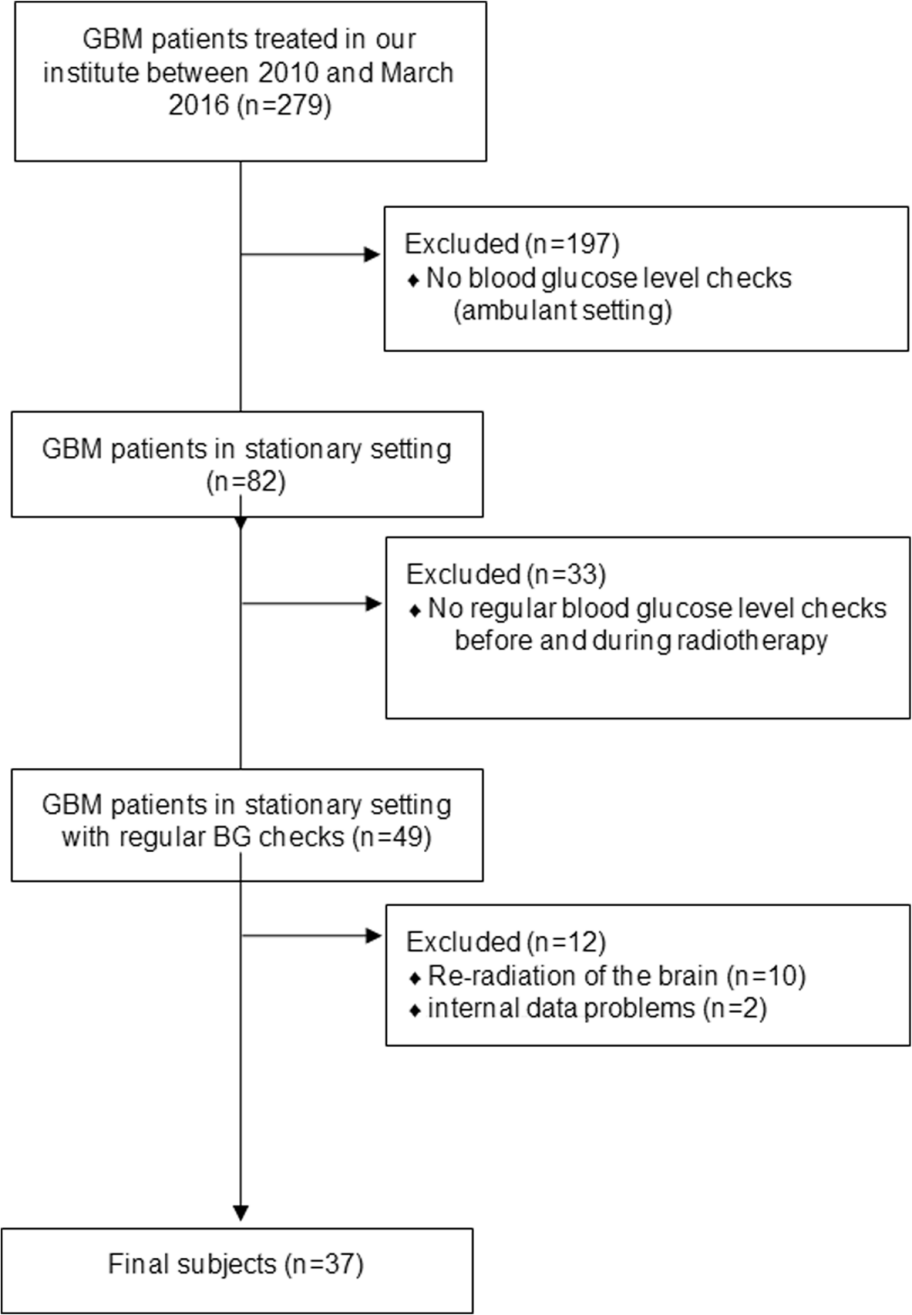
CONSORT diagram

**Fig. 2:**
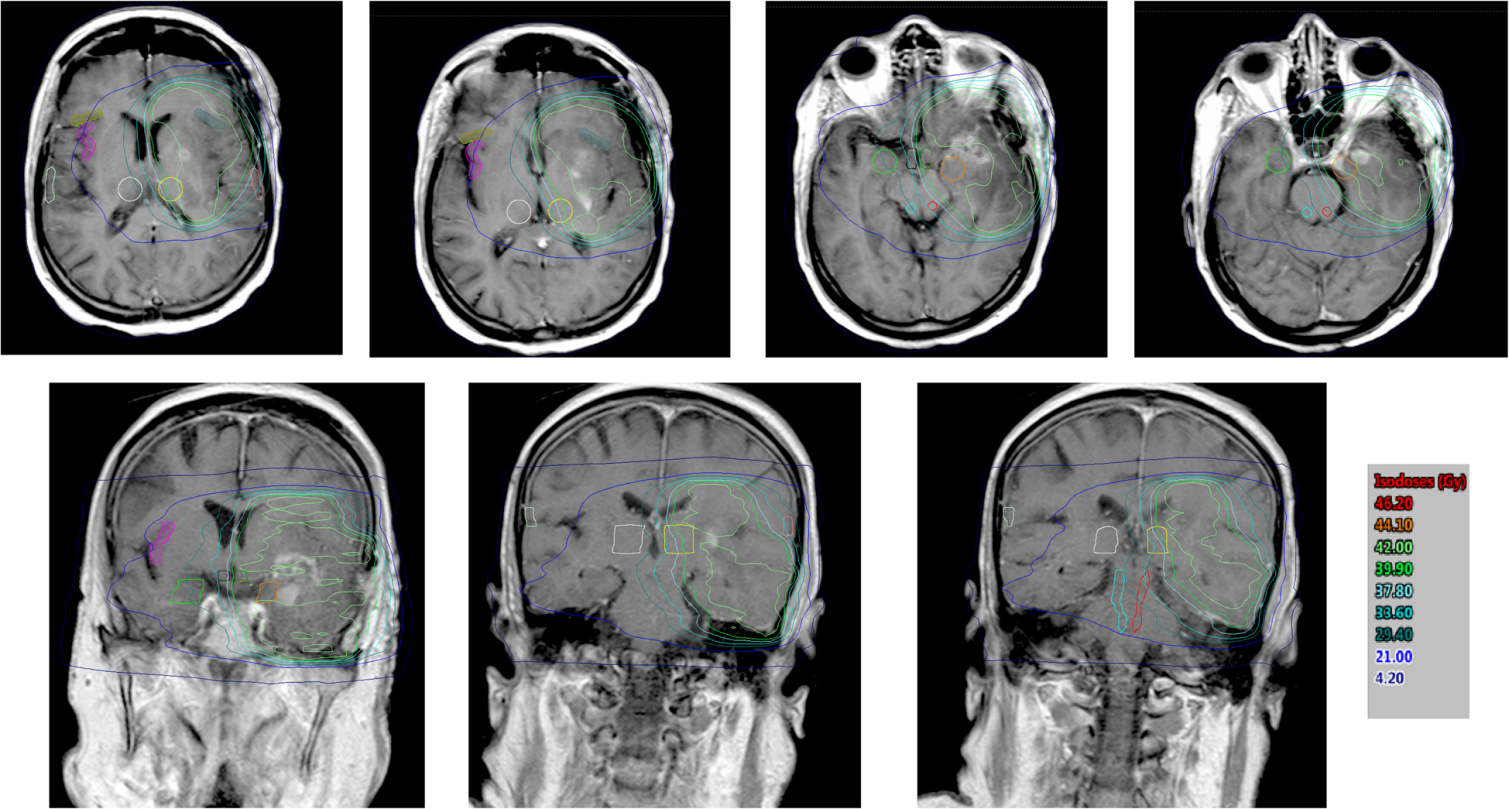
Images showing dose wash and volume delineation of the gustatory structures (light blue – right solitary tract and nucleus; red – left solitary tract and nucleus; black – right hypothalamus; dark green – left hypothalamus; white – right thalamus; yellow – left thalamus; light green – right amygdala; brown – left amygdala; pink – right anterior insula; blue – left anterior insula; olive – right frontal operculum; petrol – left frontal operculum; ice blue – right postcentral gyrus, tongue area; orange – left postcentral gyrus, tongue area)

A total of 37 patients with regular BG testing treated in our institute between 09/2010 and 03/2016 with histologically proven GBM were included into the study. Our institute treated 279 (100.0%) glioblastoma multiforme patients between 2010 and March 2016. 197 (70.6%) of them had radiation therapy in an ambulant setting, where checks of blood glucose levels were not performed. 82 (29.4%) had radiation therapy in in-patient setting. Reasons for in-patient therapy were a lower Karnofsky perfomence score. In-patients had regular blood checks. Only 49 (17.6%) had enough blood glucose measurements before and during radiation therapy to fit into the study. 10 (3.6%) of them already have had a brain radiation before. They have been excluded. With 2 (0.7%) patients we had non-congruent data A CONSORT diagram can be seen in Fig. 1.The treatment plan of these patients was re-evaluated, and distinct neuroanatomical structures were contoured (Fig. 2), and the plans were calculated. Dose levels were correlated with the clinical data. The study was approved by the Local Ethics Commission of our university (ethics number: 87/16S). A total of 22 female (59.5%) and 15 male (40.5%) patients with a median age of 64.2 years (range 27.4–85.6 years) were included. Eight patients (21.6%) out of these suffered from diabetes mellitus type 2 (DM2) (Table 1). The tumor’s locations are represented in Table 2. Most frequently the tumor is located in the right temporal lobe (*n* = 7, 18.9%). A median dose of 51.0 Gy (range 18.8–66.0 Gy) was administered in 2.0 Gy daily fractions (range 1.8–3.0 Gy per day). The radation plans and doses of our patients are quiteheterogeneous, what comes about the fact that,as said above, many of our patients were in bad physical condition. Therefore many of them needed re-evaluation during RT or quitted RT prematurely. However, most frequent combination was 30 fractions á 2.0 Gy (10 times) and a total dose of 60 Gy. The second frequent combination was the hypofractioned plan with 14 fractions á 3.0 Gy (8 times) and a total dose of 42 Gy. Eighteen patients (48.6%) got primary hypofractionated treatment. 6 patients had a re-evaluation of their initial plan during RT. Four subjects (10.8%) got a re-evaluation of their plan from curative to palliative hypofractionated treatment (three times from 2 Gy to 3 Gy, one time from 2 Gy to 2.67 Gy), 1 case was downgraded from 3 to 2 Gy single doses, and another from 2 to 1.8 Gy single doses. Two subjects (5.4%) quitted radiation earlier as planned. One patient’s plan was 66 Gy because of the boost to the primary tumor region.. A total of 16 patients (43.2%) received combined temozolomide therapy, 1 (2.7%) patient received bevacizumab, while radiation therapy alone was performed in 20 cases (54.1%). Thirty patients (81.1%) were simultaneously treated with DEXA, 1 patient (2.7%) received DEXA plus hydrocortisone in addition. DEXA was used for neurological symptoms as severe headache, nausea and vomiting, reduced vigilance or paralyzed limbs/moving disabilities. Hydrocortisone was used as substitution since the patient’s pituitary gland was destroyed by the tumor. For these patients, we assessed the daily dose of DEXA as well as the cumulative dose of DEXA during the whole RT time. We chose to report both values, as a longer and higher intake of DEXA might alter BG levels.

**Table 1:** Diabetes in patients

		female		male		Total	
		N	%	N	%	N	%
Diabetes mellitus Type 2	no	19	86.4%	10	66.7%	29	78.4%
	yes	3	13.6%	5	33.3%	8	21.6%
	Total	22	100.0%	15	100.0%	37	100.0%

**Table 2:** Tumor location

		Side of the brain							
		bilateral		left		right		Total	
		Count	N %	Count	N %	Count	N %	Count	N %
Tumor location:	multifocal	3	8.1%	1	2.7%	2	5.4%	6	16.2%
	frontal	2	5.4%	3	8.1%	5	13.5%	10	27.0%
	parietal	1	2.7%	0	0.0%	0	0.0%	1	2.7%
	temporal	0	0.0%	4	10.8%	7	18.9%	11	29.7%
	central	2	5.4%	3	8.1%	2	5.4%	7	18.9%
	fronto-temporal	0	0.0%	0	0.0%	1	2.7%	1	2.7%
	parieto-occipital	0	0.0%	1	2.7%	0	0.0%	1	2.7%
	temporo-parietal	0	0.0%	0	0.0%	0	0.0%	0	0.0%
	Total	8	21.6%	12	32.4%	17	45.9%	37	100.0%

The analyzed central gustatory structures were: solitary tract and nucleus, ventral posteromedial nucleus of the thalamus, sensory tongue area of the postcentral gyrus, anterior part of the insula, frontal operculum, amygdala and hypothalamus. These structures were delineated on magnetic resonance tomographies (MRIs) registered to planning-CTs in the Eclipse 13.0 treatment planning system (Varian Medical Systems, Palo Alto, CA, USA). All delineated structures were reviewed by a specialist in neuroradiology. In order to assure comparability between doses, all doses were transformed in equivalent doses in 2 Gy fraction (EQD2) with an α/β = 3 for the brain’s normal tissue [18]. DM2 patients were compared to non-DM2 patients and DEXA patients to non-DEXA patients. All statistical analyses were performed by using SPSS Statistics 23 (IBM, Armonk, NY, USA). Reported are average (±standard deviation) and median (range) values and *p* < 0.05 was considered as statistically significant. Comparisons between two groups were performed by t-test, regression models were used to estimate correlations among dependent and independent variables and linear correlation was used to show the strength of the relationship between variables.

## Results

**Fig. 3:**
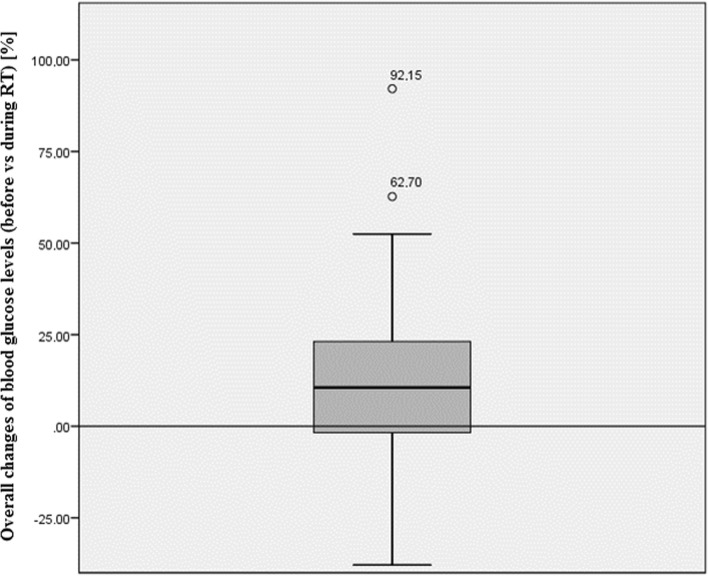
Overall changes in blood glucose compared to the levels measured before the onset of the radiotherapy

In the GBM cohort used in this study, the average daily dose of DEXA was 9.0 mg (±5.2 mg) and the total cumulative dose during the whole treatment time was 281.0 mg (±211.0). The median EQD2 dose (Dmean) to the total GS in all patients was 36.0 Gy (±8.6 Gy), 41.3 Gy (±11.1 Gy) to the ipsilateral GS structures and 31.1 Gy (±9.1 Gy) to the contralateral GS structures, respectively. Overall, glycemic control was inconspicuous during RT with an average value of 155.9 mg/dl (±47.1 mg/dl) in all cases. Nonetheless, 21 patients (56.8%) had at least one BG value over 200 mg/dl during RT. Average BG levels increased during the therapy by 14.7 mg/dl (±39.8) or + 12.8% (±25.1%), respectively (Fig. 3). Regression modelling further revealed an accelerated RT-driven increase in BG in patients that had elevated levels at the start of the radiation (b = 0.7, R^2^ = 0.411, *p* ≤ 0.001).

**Fig. 4:**
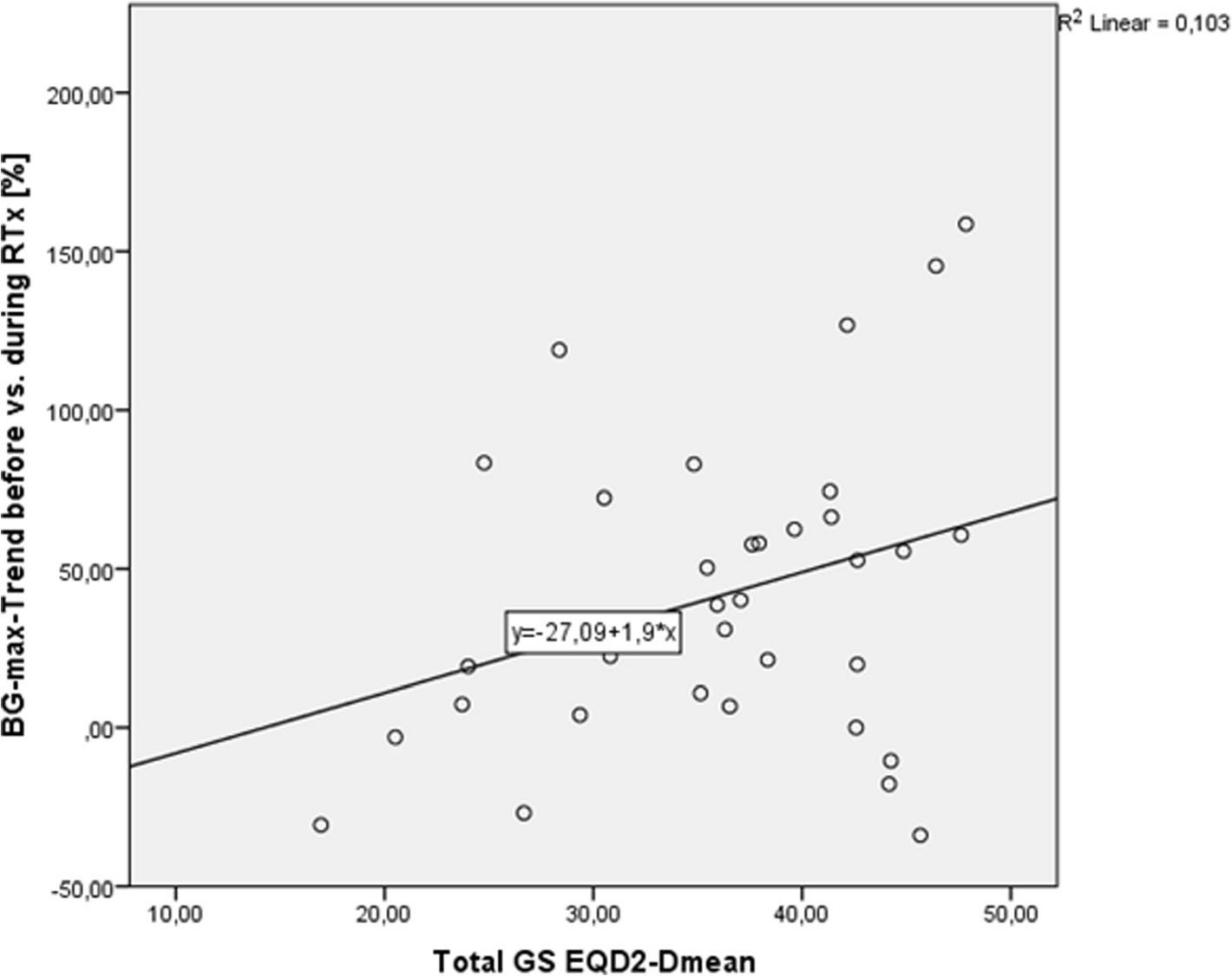
Overall BG level changes and correlation to the dose to the gustatory system

**Fig. 5:**
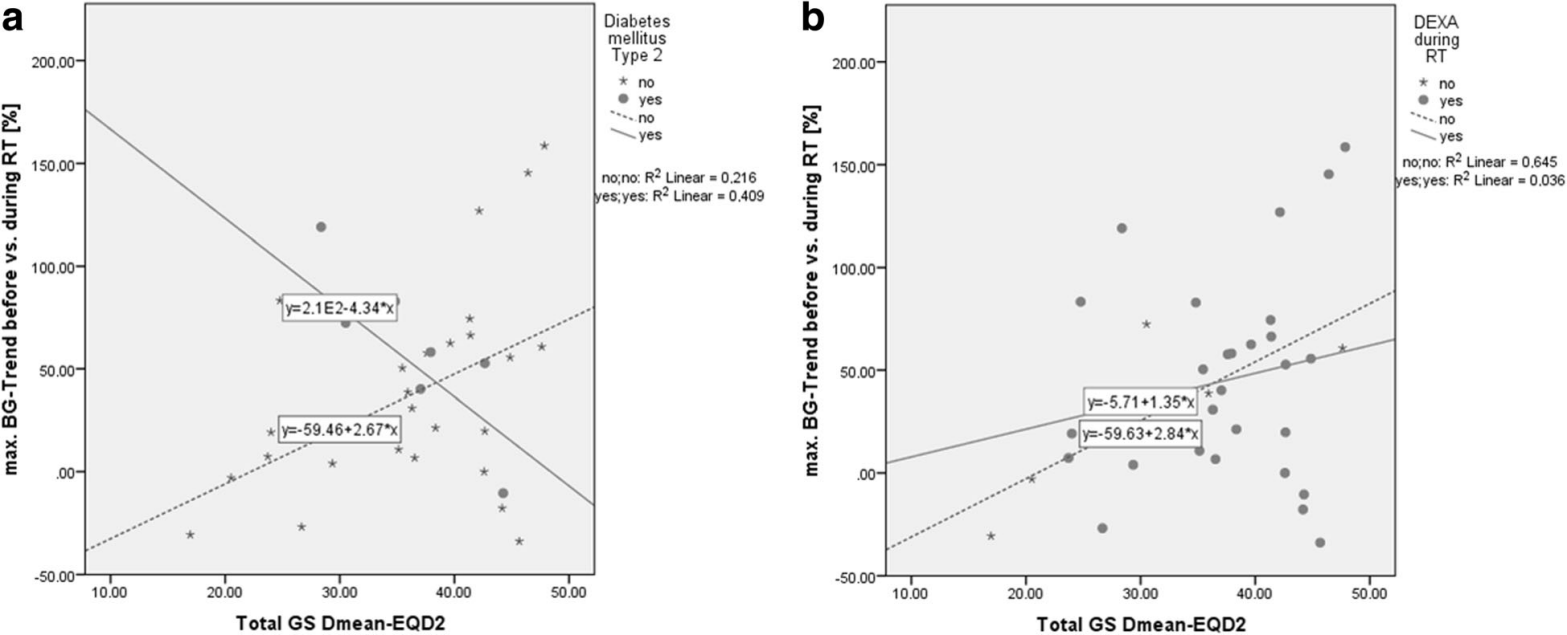
BG level changes and correlation to the dose to the gustatory system in DM2 vs. non DM2 patients (**a**) and in DEXA vs. non DEXA patients (**b**)

There was a significant difference in average BG between diabetics (192.8 ± 24.4 mg/dl) and non-diabetics (145.7 ± 39.5 mg/dl; *p* = 0.01) but no significant difference in daily DEXA medication (DM2: 7.9 ± 1.9 mg/d vs. non-DM2: 9.3 ± 5.7 mg/d; *p* = 0.29). As expected, there was a trend for increased BG in patients with DEXA-medication (162.1 ± 48.3 mg/dl) compared to patients receiving no DEXA (123.9 ± 23.2 mg/dl; *p* = 0.07). There was moreover a tendency for a larger increase in maximum BG values with more radiation dose to the total GS in the regression-model (b = 1.9, R^2^ = 0.103, *p* = 0.06) (Fig. 4). Maximum BG levels did not correlate with the daily (r = − 0.16, *p* = 0.41) and cumulative (r = 0.17, p = 0.41) DEXA therapy (Fig. 5). Four patients (10.8%) neither suffered from DM2 nor had DEXA medication. Their average BG increased by 8.1% (±5.4), which was comparable to the BG increases observed in DM2 patients (24.4 ± 56.5%; *p* = 0.59) or patients with DEXA (16.7 ± 43.4%; *p* = 0.70).

## Discussion

Our study shows a positive correlation of blood glucose levels with radiation doses applied to specific neuroanatomical regions in GBM patients. Specifically, we observed an increase in maximum BG values with higher doses applied to structures of the GS.

Our findings are consistent with recent clinical studies on survivors of acute lymphocytic leukemia (ALL) in childhood [19–21], which convincingly establish cranial RT as risk factor for metabolic disturbances such as insulin resistance and overweight. For instance, Nottage et al. [20] evaluated 784 adult ALL survivors and identified metabolic syndrome in 259 cases (33.6%) associated with prior cranial RT (RR 1.88, 95%CI 1.32–2.67). Studies on neuromolecular mechanisms for the detrimental glucoregulatory effects of RT by Marty et al. [14] pointed towards a potential role of glucose-sensing neurons in parts of the GS, especially in the nucleus of the tractus solitarious and in the hypothalamus. Lustig et al. [21] showed a dose-dependent increase in “hypothalamic obesity” after cranial irradiation of a cohort of 675 cases diagnosed and treated for primary brain tumors as children and disease-free survival greater than 5 years. Hypothalamic dosimetry greater than 51 Gy was significantly associates with a change in Body Mass Index (*p* < 0.002) [21]. The potential role of the hypothalamus in RT-mediated metabolic damage was further supported by Xu et al. [22] who experimentally investigated and described cell injury to the hypothalamus in rats in form of cell death and inflammation after a single whole-cranial irradiation of 6 Gy followed by reduced glucose tolerance.

With a Dmean of 36.0 Gy (±8.6 Gy) EQD2 to the total GS the patients in our study are far above. Our study is moreover the first which adds exact dosimetry of gustatory sub-structures such as the hypothalamus to our association analysis of GBM radiotherapy and BG. We observed an average plus of + 12.8% (±25.1%) in mean BG levels during RT and a BG increase of 1.9 mg/dl per 1 Gy to the total GS. The more pronounced the insulin resistance was before RT, the worse it got. There was a higher increase of BG in diabetics, although DEXA medication was not significantly different to non-diabetics. Our results thus resonate with the concept that cranial radiation, specifically in structures such as the hypothalamus, can impair systemic glucose control.

An average of 9 mg DEXA per day is quite high, since 1.5 mg per day is the Cushing threshold dose. As the cohort consisted of inpatients, with neurological symptoms, most of our patients needed DEXA as a treatment of headache, vigilance or speech disorders, sensomotoric deficits and other neurological complications caused by destructive tumor growth radiation and brain oedema.

The results of our study should nevertheless be interpreted with caution. Our sample size was small and the data was cross-sectional. Future studies will need larger samples and an experimental longitudinal design. Moreover, data on glycemic control of GBM patients are scarce. Barami et al. [11] did not find an association between pre-existing diagnoses such as DM2, hyperlipidemia, or obesity with the risk to develop GBM. In contrast to Barami et al. [11], who described their GBM cohort as having good glycemic control, 56.8% of the patients in our study had random BG levels ≥200 mg/dl which classifies these patients as diabetics [23]. Future studies are needed that address whether GBM per se affects glucose control in patients. In this context, it should be noted that all of our patients were inpatients. Dungan et al. [13] described the phenomenon and pathophysiology of “stress hyperglycemia” in hospital inpatients experiencing physical stress through severe illness. Elevated BG levels observed in our study may thus in part result from a complex interplay of hormones such as catecholamines, growth hormones, cortisol, and cytokines induced by the hospitalization.

## Conclusion

Our study suggests a correlation between a metabolic response and the dose to the GS. These retrospective results should be taken into consideration in the future for the development of prospective studies in RT. The knowledge about blood glucose level regulation helps to avoid complications: lower quality of life through -additional medication, food restrictions, hyperosmolar coma or worse survival. For example, the areas of the central gustatory system should be spared when planning radiation treatment as good as possible. We recommend regular blood glucose level checks on a routine basis for every patient with Glioblastoma who is undergoing radiotherapy irrespective of their hyperglycemic risk factor status.

## Data Availability

The datasets used and/or analysed during the current study are available from the corresponding author on reasonable request.

## Abbreviations

ALL: Acute lymphocytic leukemia
BG: Blood glucose
DEXA: Dexamethasone
DM2: Diabetes mellitus type 2
Dmean: Mean dose
EQD2: Equivalent doses in 2 Gy fraction
GBM: Glioblastoma multiforme
GS: Central gustatory system
MRIs: Magnetic resonance tomographies
RT: Radiation therapy
